# Omaha, 1898

**Published:** 1898-02

**Authors:** 


### OMAHA, 1898.

THE decisions of the Executive Committee of the U. S. V. M. A. to hold the meeting this year in Omaha, Nebraska, in connection with the Trans-Mississippi Exposition will call the members to a new field. It will be incumbent upon the part of the Western members to insure to the East very favorable railroad rates to secure a proper attendance of these members. The duty falling upon the few members of the Association in Nebraska and closely situated territory will be a heavy one, but ofttimes a few earnest men can do more than a large body of indifferent ones.

---

VETERINARIANS all over the land, who have watched the great struggle for freedom and independence of the brave people of Cuba, will now fully appreciate the shocking conditions that exist there—the suffering, want, loss of everything, and death of the heads of many families, and be touched to aid in the relief that should be prompt and generous from every American who appreciates what these people are struggling for. A small contribution from every one, no matter how small, will tend to diminish this sad state. Send it to your mayor or to any daily newspaper.

---

MANY meetings of State Associations will convene during February and March, and much work will be planned for the near future, all of which is of the greatest importance to the veterinary profession. Legislation will demand much time and effort, and all veterinarians should acquaint themselves with the needs of their respective States and cities, and work for the common end of higher veterinary education.

---

THE veterinarians of Ontario are thoroughly aroused to the great injustice done them by those in authority relative to the worth of examination and extermination of bovine tuberculosis. Many pertinent inquiries and urgent letters for a better recognition of the profession have gone up to the seat of government, and we are sure that their protests will not go unheeded. They should be sustained by their colleagues everywhere, and we are glad to note that some of the leading agricultural papers are in strong sympathy with the movement to place this work in competent hands only—with those educated in this work.

---

CHICAGO veterinarians have undertaken the important task of formulating rules for the examination of horses for soundness. Already many are appreciating the great scope of the subject and the many difficulties surrounding it. Already the differences in judgment are to be noted, and we fear that after all there is much to be left to one's judgment in this work, viz., acquaintance with the work the animal is to do, the price asked, and a knowledge of those who propose using it, as to how valuable our services may be, in this direction, to those who employ us.

---

We are glad to note the progress of the veterinarians of the "Windy City" toward a recognition from the Civil Service Commission charged with the selection of a City Veterinarian and the selection to be made by competitive examination. The commission of Chicago has just won a great victory in the courts as to their right to perform this work for the city. They are a body of good citizens, competent and capable, and have already given evidence of their sincerity and earnestness in this work. It is the only proper plan for the selection of any public employé.

